# The Outer Membrane Vesicles of *Aeromonas hydrophila* ATCC^®^ 7966^TM^: A Proteomic Analysis and Effect on Host Cells

**DOI:** 10.3389/fmicb.2018.02765

**Published:** 2018-11-16

**Authors:** Eric Daniel Avila-Calderón, Jorge Erick Otero-Olarra, Leopoldo Flores-Romo, Humberto Peralta, Ma. Guadalupe Aguilera-Arreola, María Rosario Morales-García, Juana Calderón-Amador, Olin Medina-Chávez, Luis Donis-Maturano, María del Socorro Ruiz-Palma, Araceli Contreras-Rodríguez

**Affiliations:** ^1^Departamento de Microbiología, Escuela Nacional de Ciencias Biológicas, Instituto Politécnico Nacional, Mexico City, Mexico; ^2^Departamento de Biología Celular, Centro de Investigación y de Estudios Avanzados, Instituto Politécnico Nacional, Mexico City, Mexico; ^3^Programa de Genómica Funcional de Procariotes, Centro de Ciencias Genómicas, Universidad Nacional Autónoma de México, Cuernavaca, Mexico; ^4^Departamento de Investigación, CICATA Querétaro, Instituto Politécnico Nacional, Querétaro, Mexico; ^5^Departamento de Innovación Biomédica, Centro de Investigación Científica y de Educación Superior de Ensenada, Ensenada, Mexico; ^6^División Químico-Biológicas, Universidad Tecnológica de Tecámac, Tecámac, Mexico

**Keywords:** outer membrane vesicles, *Aeromonas*, pathogens, Gram-negative bacteria, host cell immune response, bacterial vesicles

## Abstract

Gram-negative bacteria release outer membrane vesicles (OMVs) into the extracellular environment. OMVs have been studied extensively in bacterial pathogens, however, information related with the composition of *Aeromonas hydrophila* OMVs is missing. In this study we analyzed the composition of purified OMVs from *A. hydrophila* ATCC^®^ 7966^TM^ by proteomics. Also we studied the effect of OMVs on human peripheral blood mononuclear cells (PBMCs). Vesicles were grown in agar plates and then purified through ultracentrifugation steps. Purified vesicles showed an average diameter of 90–170 nm. Moreover, 211 unique proteins were found in OMVs from *A. hydrophila*; some of them are well-known as virulence factors such as: haemolysin Ahh1, RtxA toxin, extracellular lipase, HcpA protein, among others. OMVs from *A. hydrophila* ATCC^®^ 7966^TM^ induced lymphocyte activation and apoptosis in monocytes, as well as over-expression of pro-inflammatory cytokines. This work contributed to the knowledge of the composition of the vesicles of *A. hydrophila* ATCC^®^ 7966^TM^ and their interaction with the host cell.

## Introduction

The release of outer membrane vesicles (OMVs) from bacteria was described about 40 years ago. However, in the last 15 years much information related to these nanovesicles has been published particularly related to the composition, the biogenesis as well as with the regulation in the composition of OMVs (Beveridge, 1999; Kuehn and Kesty, 2005; Mashburn-Warren and Whiteley, 2006; Lee et al., 2008). In general, OMVs possess a bilayer membrane containing lipoproteins, outer membrane proteins (OMP), lipopolysaccharide (LPS), periplasmic and cytoplasmic proteins. Butmore recently, many studies based on mass spectroscopy analysis have been performed to learn more about the components of these vesicles in both, pathogenic and nonpathogenic bacteria (Lee et al., 2008). OMVs have been implicated in many processes including the release of virulence factors such as proteases and toxins, molecules signaling between bacterial and eukaryotic cells, DNA transfer, antibacterial activity, immunomodulation of the host and facilitation of bacterial survival during envelope stress (Kuehn and Kesty, 2005; Mashburn-Warren and Whiteley, 2006; Lee et al., 2008). Virulence factors have been identified in bacterial pathogens; for instance the heat/labile enterotoxin (LT), the Shiga toxins 1 and 2, as well as the ClyA cytotoxic protein found in the OMVs of *Escherichia coli* (Gankema et al., 1980; Wai et al., 1995, 2003; Kolling and Matthews, 1999). Also the cholera toxin (CT) of *Vibrio cholerae* was associated with OMVs (Chatterjee and Chaudhuri, 2011) meanwhile *Helicobacter pylori* OMVs contain the cytotoxins VacA, and CagA (Olofsson et al., 2010).

One particular feature of OMVs is their ability to disseminate through the host cell, delivering virulence factors to different organs. Due to the nanostructure of OMVs, these vesicles reach tissues even more deeply than whole bacteria (Kulp and Kuehn, 2010). In this context, purified OMVs from *E. coli* administered intraperitoneally in mice caused inflammation in the lungs, showing their ability to disseminate (Jang et al., 2015). Moreover, OMVs have been implicated in sepsis, delivering endotoxin (LPS) to the endothelial cells, inducing the production of pro-inflammatory cytokines and upregulating the expression of adhesion molecules, facilitating the transition from localized to systemic infection (Soult et al., 2013).

For instance, PBMCs stimulated with OMVs from *H. pylori* induced the expression of cyclo-oxygenase 2 (COX-2) and IL-10 from monocytes both suppress T cell response. Thus, *H. pylori* not only modulate local immune response but also could modulate peripheral immune response (Hock et al., 2017). Moreover, OMVs from *E. coli* were up-taking by gastric epithelial cells (Caco-2), OMVs induced the secretion of cytokines which subsequently activated PBMCs, these results showed that OMVs are able to signal through the mucosal barrier (Fábrega et al., 2016).

Naturally during the infection of the host, the Gram-negative pathogens release OMVs; i.e., *Legionella pneumophila* released OMVs inside the phagosomes of *Dictyostelium discoideum* after infection (Shevchuk et al., 2011). Inside the host, Gram negative pathogens face several mechanism and harsh conditions imposed by the immune response. It has been proposed that the over-production of the OMVs may helps the bacteria to survive under harsh conditions in the host; for instance, Enterotoxigenic *E. coli* increased the production of the OMVs after the passage through the mouse intestine (Ellis and Kuehn, 2010).

*Aeromonads* are Gram negative natural waters inhabitants, have been associated with a wide spectrum of fish and human diseases. The genus *Aeromonas* taxonomy is continuously changing, currently 36 species have been taxonomic proposed. However, few of these are etiological agents of skin and soft-tissue infections, and gastroenteritis. In addition, these organisms have been recognized as a cause of foodborne and waterborne outbreaks of diseases (Janda and Abbott, 2010).

Several virulence factors have been associated with pathogenicity of clinical as well as environmental *Aeromonads* strains. The main pathogenic factors associated with *Aeromonas* are: surface polysaccharides (capsule, lipopolysaccharide, and glucan), S-layers, iron-binding systems, exotoxins and extracellular enzymes, secretion systems, fimbriae and other nonfilamentous adhesins, motility and flagella (Tomás, 2012). All of them are regarded as the multifactorial mechanisms of pathogenicity. However, the ability to produce these factors is not uniform in all the isolates. The most common virulence factor is the expression of two main types of enterotoxins: the cytotoxic and the cytotonics ones. Type III and VI secretion systems (T3SS and T6SS, respectively) have been documented to play a critical role in the virulence of many Gram-negative bacteria including *Aeromonas*, are often activated upon contact with target cells and deliver their toxin proteins, the so-called effectors, directly into the host cells cytosol. Additionally, in *Aeromonas* there are mechanisms that are associated with host cell apoptosis/pyroptosis for instance *Aeromonas* T2SS secreted Act, a cytotoxic enterotoxin, and *Aeromonas* T6SS secreted the effector hemolysin co-regulated protein (Hcp) (Rosenzweig and Chopra, 2013).

To the best of our knowledge only few studies have described the presence of extracellular vesicles elements in *Aeromonas*. However, these only have described ultrastructural and morphological aspects (Longa-Briceno et al., 2008; Lusta and Kozlovskii, 2011). To date the protein composition of OMVs from *A. hydrophila* has not been yet explored. In the attempt to increase the understanding of the composition of these vesicles, the proteome of OMVs isolated from *A. hydrophila* ATCC^®^ 7966^TM^ is described. Also, we analyzed the ability of the OMVs to regulate expression of activation/inhibition surface markers and apoptosis induction in peripheral blood mononuclear cells, as well as cytokine production.

## Materials and Methods

### Purification of OMVs

The *A. hydrophila* ATCC^®^ 7966^TM^ strain was identified by the multilocus phylogenetic analysis (MLPA) proposed by Martinez-Murcia et al., 2011.

The OMVs purification was performed according to the protocol described by Avila-Calderón et al. (2012). Briefly, *A. hydrophila* ATCC^®^ 7966^TM^ was cultured massively on tryptic soy agar (TSA) (Becton Dickinson-BD^TM^) plates incubated 24 h at 37°C. Then bacteria were harvested with a rubber policeman and suspended in 250 ml sterile PBS 0.1 M (Gibco^®^). The bacterial suspension was centrifuged at 10,000 × *g* for 30 min (Thermo Scientific^TM^ Sorvall^TM^ Legend^TM^ XT/XF.) The supernatant was passed through a 0.22 μm filter (Millipore Corp.); and a sterility test was performed by culturing an aliquot onto a TSA plate followed by incubation for 24 h at 37°C. The sterile supernatant was centrifuged at 100,000 × *g* for 2 h at 4°C (Beckman Coulter Optima L-90K). The pellet was washed twice with 25 mL of sterile PBS and the OMVs were suspended in 1 mL of sterile PBS (raw OMVs). The total protein concentration was determined using PIERCE-BCA (Thermo Fisher Scientific Inc.) reagents as per manufacturer’s recommendations. The OMVs samples were divided into 0.5 mL aliquots and stored at -20°C until used.

### Gradient Ultracentrifugations

Outer membrane vesicles were purified with density gradient using OptiPrep (Sigma-Aldrich, Inc.) according to the protocol of Fernandez-Moreira et al. (2006). Briefly, OptiPrep was diluted with sterile PBS at final concentrations of 10, 15, 20, 25, and 30 %. 2.6 mL of OptiPrep solutions were layered sequentially in an ultracentrifuge tube from the higher to the lower density. OMVs were loaded at the bottom of the tube. Tubes were centrifuged at 100, 000 × *g* for 16 h at 4°C. OMVs appeared as an opalescent band in the density gradient. OMVs were collected, washed twice with sterile PBS at 100, 000 × *g* during 2 h at 4°C and finally suspended in 300 μL of PBS.

### Electron Microscopy

Twenty microliter of purified OMVs (25 μg of protein) were placed onto copper grids coated with formvar and dried using filter paper. 1% phosphotungstic acid was added and the grids were allowed to stand overnight at room temperature; they were observed under the transmission electron microscope (JEOL model JEM 10-10).

To observe vesicles released from bacteria, *A. hydrophila* ATCC^®^ 7966^TM^ was grown in TSA plates overnight, and then molten soft agar was poured over growth. Once solidified the agar, small cubes (2 mm) of agar were cut. Blocks were fixed in glutaraldehyde 2.5% in PBS, rinsed with Sorensen’s PBS, dehydrated with ethanol, and prepared to transmission electron microscopy (TEM). All preparations were stained with OsO_4_, and they were observed in transmission electron microscope (JEOL model JEM 10-10).

### Denaturing Gel Electrophoresis

MOPS-SDS-PAGE was performed in 12.5% acrylamide slab gels by the method of Laemmli. The gels were stained with Coomassie blue. The apparent molecular masses purified OMVs proteins were determined by comparing their electrophoretic mobility with that of the wide range molecular mass markers (SigmaMarker^TM^; Sigma-Aldrich, Inc.) using the computer program SigmaGel^®^ V. 1.0.

### LC-MS/MS

After the separation of purified OMVs by denaturing electrophoresis, the acrylamide gel was cut into four sections. Each section of the gel was treated with 50 mM dithiothreitol, alkylated with iodoacetamide and then “in gel” digested with trypsin. The peptides were desalted using a ZipTip^®^ (Merck KGaA, Darmstadt, German) and then concentrated in a Speed-Vac SPD 1010 Thermo Electron (Instituto Nacional de Biotecnología-UNAM, Cuernavaca, México).

The samples were dissolved with 50% acetonitrile containing 1% acetic acid and then placed into a Finnigan LCQ equipment. The eluate at 10 L/min was split to allow only 5% of the sample to enter the nanospray source (0.5 L/min). LC MS/MS was performed with a PicoFrit needle/column RP C18 (New Objective, Woburn, MA, United States), using a fast gradient system from 5 to 60% of solution 100% acetonitrile with 1% acetic acid, for 45 min. The electrospray ionization source voltage was set at 1.8 kV and the capillary temperature at 130°C. Collision-Induced Dissociation (CID) was performed using 25V of collision energy, 35–45% (arbitrary units) of normalized collision energy and the scan had the wide band activated. All spectra were obtained in the positive-ion mode. Data acquisition and the deconvolution of data were carried out using Xcalibur software on a Windows XP PC system. The MS/MS spectra from enzymatically generated peptides were analyzed by Sequest software from Finnigan (Palo Alto, CA, United States) and MASCOT search engine from Matrix Science Ltd (Boston, MA, United States).

### *In silico* Analysis

The hits from the proteomic identification were analyzed by BlastP, using the genomic sequence of the *A. hydrophila* ATCC^®^ 7966^TM^ (obtained from NCBI^1^). String was used to find metabolic networks associated to the proteins with hypothetical function (string.embl.de). Evpedia analysis for enrichment of gene ontology terms was also used http://student4.postech.ac.kr/evpedia2. Each protein with hypothetical function was searched in PSORTb v. 3. 0. From ExPASy Bioinformatics Resource Portal^2^ and ProtCompB from Softberry database^3^ were used to subcellular location of each protein.

### OMVs Stimulation *in vitro*

#### PBMCs Isolation

Peripheral blood mononuclear cells were isolated from buffy coats from healthy donors. Briefly, blood units were diluted with sterile PBS and layered into centrifuge tube with equal volume of Ficoll-Paque Premium (GE Healthcare). Blood was centrifuge at 2, 000 × *g* during 25 min at room temperature, and washed twice. Then cells were counted in a Neubauer chamber with Trypan blue. Cells were adjusted at concentration of 1 × 10^6^ /mL, in RPMI (Gibco^®^) supplemented with 10% FCS, 50 μg/mL gentamicin (Gibco^®^) and 2.5 μg/mL Fungizone (Gibco^®^). PBMCs were plated at 1 × 10^6^ /mL/well in a 24-well plate. Healthy donors gave their written consent to participate in this publication.

### Evaluation of Surface Markers of Peripheral Blood Mononuclear Cells (PBMCs) Induced by OMVs

Formalin-killed bacteria (FKB) was obtained as follows: *A. hydrophila* ATCC^®^ 7966^TM^ was cultured 24 h onto TSA plates, then a cell suspension was prepared in sterile phosphate-buffered saline (PBS 0.1 M, pH 7.3). A serial dilution was performed to reach 10^10^ bacteria/mL. Bacterial suspension was centrifuged, PBS was discarded and the pellet was resuspended with 2 mL PBS-Formalin 1%, and incubated 5 min. Bacterial suspension was washed 2 times with sterile PBS, and restored with 1mL of sterile PBS. This last cell suspension was used as FKB.

PBMCs were stimulated with 10 or 25 μg/mL of OMVs, FKB at MOI 1:10, or 5 μg phytohaemagglutinin (PHA; Gibco^TM^) during 12, 24, and 48 h. Plates were incubated at 37°C with 5% CO_2_. Monoclonal antibodies (mAbs) coupled to fluorochromes: anti-human CD91-eFluor 660 (eBioscience), anti-human CD3-APC (BD), anti-human CD19-PE-Cy7 (BD), anti-human PD-L1-PE (BioLegend), anti-human PD-1-FITC (BioLegend), anti-human CD86-PE (BioLegend), anti-human CD69-TRI-COLOR (Molecular Probes) were used. After stimulation, 100 μL of supernatant were collected from the wells and stored at -70°C until use. Also, PBMCs were collected and washed with FACS buffer (PBS, 1% FCS, sodium azide 0.01%) and incubated with diluted mAbs in FACS buffers during 1 h at 4°C protected from the light. Then cells were washed with FACS buffer, fixed with PBS 1% paraformaldehyde (PFA), and finally resuspended in 400 μL of FACS buffer. Samples were analyzed in LSRFortessa^TM^ cytometer (DB), 30,000 events were acquired and data were analyzed with FlowJo^®^ software v. 10 (FlowJo, LLC, Ashland, OR, United States).

### Cytokine Quantification

Cytokine quantification of inflammatory cytokines; IL-8, IL-1β, IL-6, IL-10, TNFα, and IL-12p70 was performed with CBA Human inflammatory cytokine kit (BD Biosciences San Diego, CA, United States) from supernatants collected according manufacturer’s instructions. Samples were read in LSRFortessa^TM^ cytometer (BD Biosciences) with CBA template, 2000 events were collected and data were analyzed with FCAP Array software v. 3. 0 (BD Biosciences).

### Apoptosis, DNA Damage and Cell Proliferation Induced by OMVs

Apoptosis, DNA Damage and Cell Proliferation Kit (BD Pharmingen^TM^) was used to evaluate apoptosis, DNA damage, and cell proliferation. PBMCs were stimulated with 10 or 25 μg/mL of OMVs or 10 μL of 1 mM Carbonyl cyanide 3-chlorophenylhydrazone (CCCP; Abcam) during 24 h. Then BrdU was added to the cells an incubated during 1 h at final concentration of 10 μM, then cells were collected, washed with FACS buffer, and staining with mAbs according manufacturer’s instructions. Anti-BrdU-PerCP-Cy5.5, anti-human H2AX-Alexa Flour 647, and anti-cleaved PARP-PE. PARP [Poly (ADP-Ribose) Polymerase] is a nuclear chromatin-associated enzyme that is involved in DNA repair. During apoptosis, Caspase-3 cleaves PARP resulting in its inactivation and the inability of cells to repair DNA damage. For this reason, the 89 kDa-cleaved fragment of PARP serves as a marker of cellular apoptosis. 30,000 events were acquired in LSRFortessa cytometer (BD Biosciences), and data were analyzed with FlowJo software v. 10 (FlowJo^®^, LLC, Ashland, OR, United States).

### PBMCs Treated With OMVs

1 × 10^6^ PBMCs were plated on sterile cover slips and stimulated with 10 μg/mL of OMVs or PHA during 24 h. After that, cells were fixed with increasing concentration of PBS plus PFA (0.5, 1, and 2%) during 30 min. Cells were stained with Giemsa diluted 1:10 with phosphates solution during 10 min, and rinsed with water. PBMCs were observed with a bright-field BX51 microscope (Olympus).

### Effect of OMVs on PBMCs Cytoskeleton

PBMCs were fixed onto cover slips by duplicated and permeabilized with Triton X-100 0.5% in PBS. Then, one set of cells were treated with PBS/BSA 1% plus human serum 1% during 1 h at room temperature, and later washed with PBS/BSA 0.2%. After that, cells were incubated with 50 μL of mAb anti-tubulin (Biolegend) diluted 1:100 in PBS/BSA 1%, overnight at 4°C in a humid chamber. Cover slips were washed three times and incubated with mAb goat anti-mouse IgG Alexa fluor-488 (Invitrogen^TM^, Thermo Fisher Scientific Inc.) during 1 h at room temperature, in the darkness. On the other hand, the other one set of cover slips with fixed cells were incubated with rhodamine-phalloidin (Molcular Probes^TM^) diluted 1:100 in PBS during 30 min in the darkness. Both sets of cover slips were washed, and stained with DAPI 1 mg/mL diluted 1:10 in PBS during 1 min. Finally, cover slips were washed and mounted with DABCO solution. Cells were observed in a confocal microscopy Leica TCS SP8 AOBS (ACOUSTO-OPTICAL BEAM SPLITTER) DMI6000 (Leica Microsystems, Germany). At least three fields were captured for the analysis.

### Ethics Statement

The present work was approved by the ethic committee of Escuela Nacional de Ciencias Biológicas Instituto Politécnico Nacional approval number 4265. A written informed consent was signed by the healthy donors.

### Statistics

Two-way ANOVA analysis with Bonferroni post-tests was performed for surface markers expression and cytokine production, and One-way ANOVA analysis with Tukey post-test was used to compare results in other experiments. GraphPad Prism V. 5. 01 and SigmaStat V. 4. 0., software was used for the statistical analysis.

## Results

### Purification of OMVs From *Aeromonas hydrophila* ATCC^®^ 7966^TM^

**FIGURE 1:**
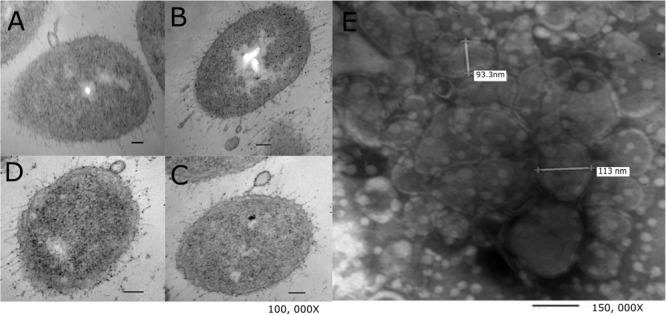
Electron microscopy of *A. hydrophila* ATCC^®^ 7966^TM^ OMVs. **(A–D)** Agar embedded whole bacteria was processed for thin section and negative stained with OsO_4_; in the micrographs OMVs are pinch off from the bacterial surface (magnification 100, 000 ×). **(E)** Purified OMVs stained with fosfotungstic acid showed vesicles with a double membrane ranging in size from 90 to 100 nm (magnification 150, 000 ×). Bars = 100 nm.

*Aeromonas hydrophila* ATCC^®^ 7966^TM^ whole cell showed a vesicle still attached to the outer membrane, but also smaller vesicles together one after the other, forming prayer beads (Figure 1A). Also, we could observe vesicles releasing from several cells (Figure 1B). In the micrograph, the vesicle pinched off from the cell with a tendril attached to the membrane. Vesicles showed a size in average near 100 nm (Figures 1C,D). In Figure 1E we can observed the purified vesicles after to apply an Optiprep gradient.

### SDS–PAGE and Proteomic Analysis

**FIGURE 2:**
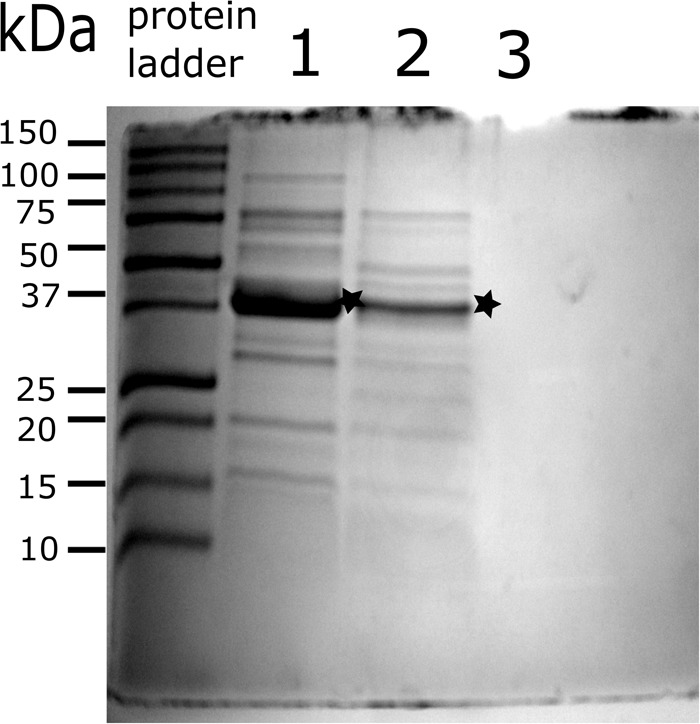
Protein profile of *A. hydrophila* ATCC^®^ 7966^TM^ OMVs. OMVs were purified by differential centrifugation and loaded onto 12.5% Acrylamide gel for electrophoresis. Lane 1; purified OMVs by ultracentrifugation at 150, 000 × *g*, during 2 h. Lane 2; OMVs purified by Optiprep gradient centrifuged at 150, 000 × *g*, during 16 h. 25 μg of protein of OMVs were loaded on each well. Lane 3; PBS from the second step-wash of purified vesicles. ^∗^Protein of approximately 42 kDa which could correspond to flagellar body (FlaA/FlaB).

OMVs from *A. hydrophila* ATCC^®^ 7966^TM^ showed an electrophoretic protein profiles ranging from 10 to 120 kDa proteins (Figure 2). Then, “in gel” digested with trypsin was performed to know the protein composition of vesicles. The trypsin generated peptide masses, as well as their fragment ions, were analyzed by LC-MS/MS. The resulting peptides sequences were used to query databases that led to the identification of 211 unique proteins. A query result was only considered as significant if the overall score was higher than 25 and more than two tryptic peptides as well as their fragment ions matched to the protein and the calculated molecular weight corresponded to molecular weight in the original gel section.

The Unit reported 264 hits. These hits were analyzed with BlastP using the genome of *A. hydrophila* subsp. *hydrophila* strain ATCC^®^ 7966^TM^. The hits that were unambiguosly identified in the genome were 211, with *p* < 1 × 10^-4^. The rest of the sequences were discarded for further analysis. Using the COG functional classification, 191 belonged to known functional classes and 36 had not a COG class (Supplementary Table S1). According to COG classification, proteins identified in *A. hydrophila* OMVs were classified into: proteins involved in metabolism, cellular processes and signaling proteins, information, storage and processing proteins and poorly characterized proteins. Besides, the *in silico* analysis revealed that 69 proteins (32.7%) were from cytoplasmic location, 52 (24.6%) from inner membrane, 39 (18.5%) from outer membrane, 32 (15.2%) from periplasmic location and 19 (9%) were extracellular proteins.

The Supplementary Figure S1 shows the comparison between the *A. hydrophila* ATCC^®^ 7966^TM^ genome and the proteins found in the vesicles. In this figure, we can see that proteins contained in the vesicles had several functions, such as cell wall synthesis, cell motility, intracellular trafficking, and amino acid, lipid and inorganic ions metabolism and transport. On the other hand, functions comparatively enriched in the genome were transcription, replication, signal transduction, coenzyme metabolism and general function. Interestingly, translation was a function with identical proportion of proteins in the genome and vesicles.

The presence of some proteins was remarkable. For example, 14 outer membrane proteins, flagellin, extracellular lipases, hemolysins, pullalanase, HcpA protein, RtxA toxin and proteins from polar flagella (FlaA/FlaB). An analysis for enrichment of gene ontology terms in biological processes showed significant overrepresentation of response to metals (cadmium) and inorganic substances, and metabolism of hexose, monosaccharide, glucose, carboxilic acid and oxoacids. Regarding metabolic function, proteins with the activities of exopeptidases, serine-peptidases, hydrolases, siderophore, and acyl carriers transport were enriched in vesicles. Using String, we obtained functional relationships for 6 proteins with hypothetical function: AHA_2808, AHA_3399, AHA_1832, AHA_1554, AHA_2419, and AHA_3636 (Supplementary Table S1).

Some putative proteins related to antibiotic resistance like AcrB (AHA_291; COG0841) or LppC superfamily protein (AHA_3895; COG3107) were identified. This putative role was assigned by the ontology classification. Further investigation to assess the role of these proteins in antibiotic resistance is needed.

### *A. hydrophila* ATCC^®^ 7966^TM^ OMVs Induce Lymphocyte Activation and Expression of PDs Molecules

**FIGURE 3:**
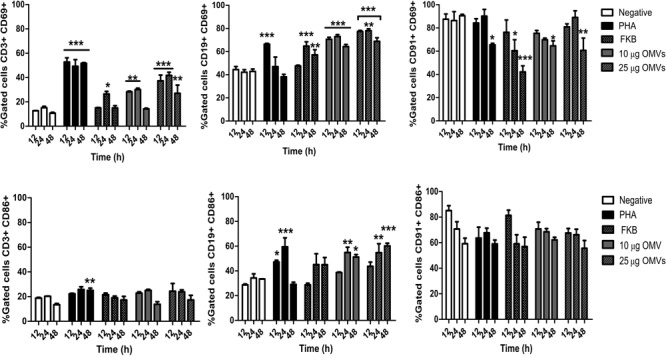
Analysis of expression of activation surface markers in PBMCs stimulated with *A. hydrophila* ATCC^®^ 7966^TM^ OMVs. PBMCs from healthy donor were co-cultured with OMVs from *A. hydrophila*. Activation was measured by flow cytometry using mAbs against surface molecules (CD69, CD86). Monocyte (CD91+) and lymphocytes (CD3+, CD19+) were gated to analyze individually the expression of surface molecules. ^∗^*P* < 0.05, ^∗∗^*P* < 0.01, ^∗∗∗^*P* < 0.001.

To analyze the expression of surface markers in PBMCs, 10 and 25 μg/mL OMVs were used to stimulate cells at different time points. These results were compared with the effect of the mitogenic compound PHA, or unstimulated cells as a negative control. To differentiate the populations in PBMCs mAbs anti human CD3+ (T lymphocyte), CD19+ (B lymphocytes) and CD91+ (monocytes) were used as reported previously (Hudig et al., 2014). Both concentrations of *A. hydrophila* ATCC^®^ 7966^TM^ OMVs (10 and 25 μg/mL) induced expression of significantly high levels of the early activation marker CD69+ in both populations of lymphocytes; CD19+ and CD3+ cells at different time points compared with unstimulated cells (^∗∗^*P* < 0.01, ^∗∗∗^*P* < 0.001). Moreover, over-expression of CD69 was higher in CD19+ than in CD3+ cells, and higher than in cells stimulated with PHA or FKB (Figure 3). However, no significant expression of CD69 was found in monocytes (CD91+) at late time (48 h), conversely CD69 expression decreased in CD91+ cells compared with unstimulated cells. *A. hydrophila* OMVs only induced high expression of CD86 in LnB CD19+ at late times (24 and 48 h) at similar levels than in cells stimulated with PHA, but not in monocytes (CD91+) or CD3+ cells (Figure 3). No significant expression of CD86 was found in cells stimulated with FKB.

**FIGURE 4:**
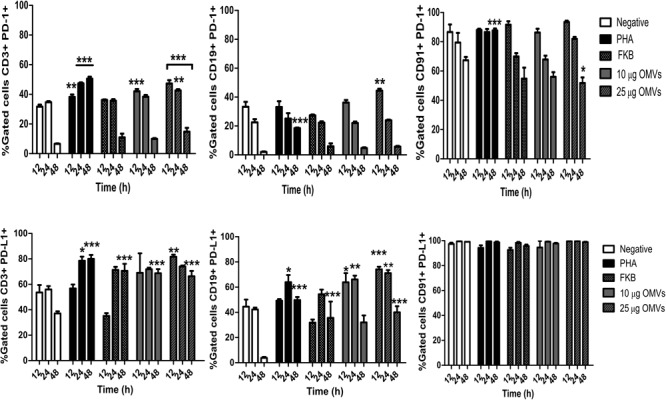
Analysis of expression of inhibition surface markers in PBMCs stimulated with *A. hydrophila* ATCC^®^ 7966^TM^ OMVs. PBMCs from healthy donor were co-cultured with OMVs from *A. hydrophila*. Inhibition was measured by flow cytometry using mAbs against surface molecules (PD-1, PD-L1). Monocyte (CD91+) and lymphocytes (CD3+, CD19+) were gated to analyze individually the expression of surface molecules. FKB were used as control for the expression of surface markers. ^∗^*P* < 0.05, ^∗∗^*P* < 0.01, ^∗∗∗^*P* < 0.001.

On the other hand, *A. hydrophila* OMVs increased the expression of programed death-1 molecule (PD-1) only in LnT CD3+ at 12 and 24 h post-stimulation at similar levels than PHA-stimulated cells (^∗∗^*P* < 0.01, ^∗∗∗^*P* < 0.001). Furthermore, both concentrations of OMVs induced high levels of programed death-ligand-1 (PD-L1) similar those induced by PHA and FKB in CD3+ cells. The expression of PD-L1 in CD19+ cells was little higher in those cells stimulated with both concentrations of OMVs at 12 and 24 h post-stimulation than in cells stimulated with PHA or FKB (^∗∗^*P* < 0.01, ^∗∗∗^*P* < 0.001). No significant expression of PD-1 or PD-L1 was observed in monocytes stimulated with OMVs, PHA, or FKB (Figure 4).

### *A. hydrophila* ATCC^®^ 7966^TM^ OMVs Induce Inflammatory Cytokine Profile in PBMCs

**FIGURE 5:**
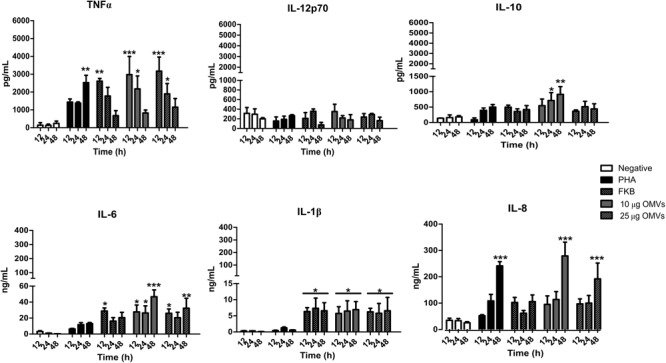
Cytokine quantification from PBMCs stimulated with *A. hydrophila* ATCC^®^ 7966^TM^ OMVs. Inflammatory cytokines were determined by CBA cytometric assay at different time points. *A. hydrophila* OMVs induced high levels of inflammatory cytokines such as IL-8, IL-1β, IL-6, and TNFα. ^∗^*P* < 0.05, ^∗∗^*P* < 0.01, and ^∗∗∗^*P* < 0.001.

To shed light on the underlying mechanisms of the response elicited by PMBCs stimulated with OMVs, we analyzed the cytokine profile. PBMCs stimulated with OMVs induced levels of IL-6 higher than those induced by PHA or FKB (^∗^*P* < 0.05, ^∗∗^*P* < 0.01, and ^∗∗∗^*P* < 0.001). Also, both OMVs induced the production of TNFα at 12 and 24 h post-stimulation, lower levels were observed with PHA or FKB (^∗^*P* < 0.05, ^∗∗∗^*P* < 0.001), whereas at 48 h TNFα decreased. IL-10 production was higher in PBMCs stimulated with 10 μg/mL of OMVs (^∗^*P* < 0.05, ^∗∗^*P* < 0.01) at 24 and 48 h post-stimulation. Not significant IL-10 production was observed with PHA, FKB or 25 μg/mL OMVs. Moreover, both concentrations of OMVs and FKB induced significantly (*^∗^P* < 0.05) the highest concentration of IL-1β (ng/mL), while PHA and both concentration of *A. hydrophila* OMVs induced significantly the highest concentration of IL-8 at 48 h post-stimulation (^∗∗∗^*P* < 0.001) (Figure 5). No significant production of Il-12p70 was observed with the different stimulus tested.

### Monocytic Cells Stimulated With *A. hydrophila* ATCC^®^ 7966^TM^ OMVs Over-Express Cleaved PARP

**FIGURE 6:**
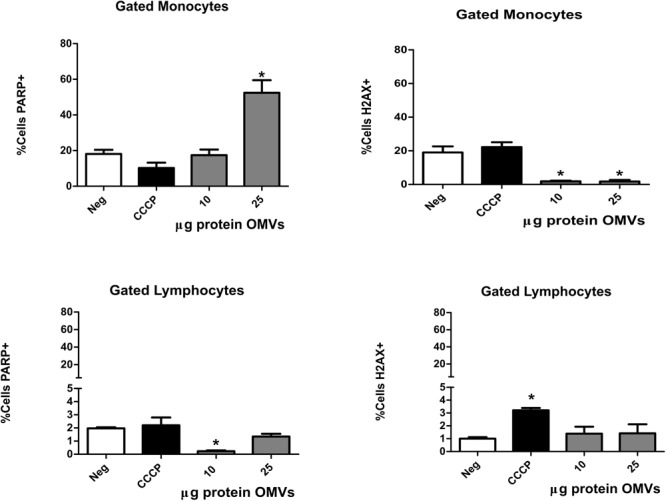
Apoptosis and DNA damage of PBMCs induced by *A. hydrophila* ATCC^®^ 7966^TM^ OMVs. PBMCs from healthy donor were co-cultured with different concentrations of *A. hydrophila* OMVs during 24 h. Apoptosis and DNA damaged was evaluated by flow cytometry with mAbs anti-PARP and anti-H2AX. CCCP was used as control for apoptosis induction. ^∗^*P* < 0.05.

To determine whether OMVs were biologically active in host cells, we analyzed proliferation, DNA damage and apoptosis in PBMCs stimulated with *A. hydrophila* OMVs. PBMCs were stimulated with 10 and 25 μg/mL OMVs during 24 h, then a multiparametric analysis by flow cytometry was performed. OMVs did not induce DNA synthesis measured with anti-BrdU in PBMCs (data not shown). Also, DNA damage was analyzed by the expression of phosphorylated histone H2AX; interestingly *A. hydrophila* OMVs decreased significantly the expression of H2AX histone in the monocyte region (Figure 6). The percentage of cells H2AX^+^ from the lymphocyte region slightly increased when cells were stimulated with OMVs, but not significantly compared with unstimulated CCCP control (Figure 6). On the other hand, apoptosis was evaluated by measuring the cleaved PARP [Poly (ADP-ribose) polymerase-1]. Cells from lymphocyte region did not show significant increase in the level of cleaved PARP when cells were stimulated with OMVs. Only cells from the monocyte region stimulated with 25 μg/mL of OMVs showed significantly higher levels of cleaved PARP (*P* < 0.05). These results suggested that higher concentrations of OMVs induced apoptosis via cleavage of PARP in monocyte but not in lymphocytes. Besides, OMVs did not induce the expression of H2AX histone or DNA synthesis in PBMCs.

### *A. hydrophila* ATCC^®^ 7966^TM^ OMVs Affects Morphological Shape and Cytoskeleton Structure of PBMCs

**FIGURE 7:**
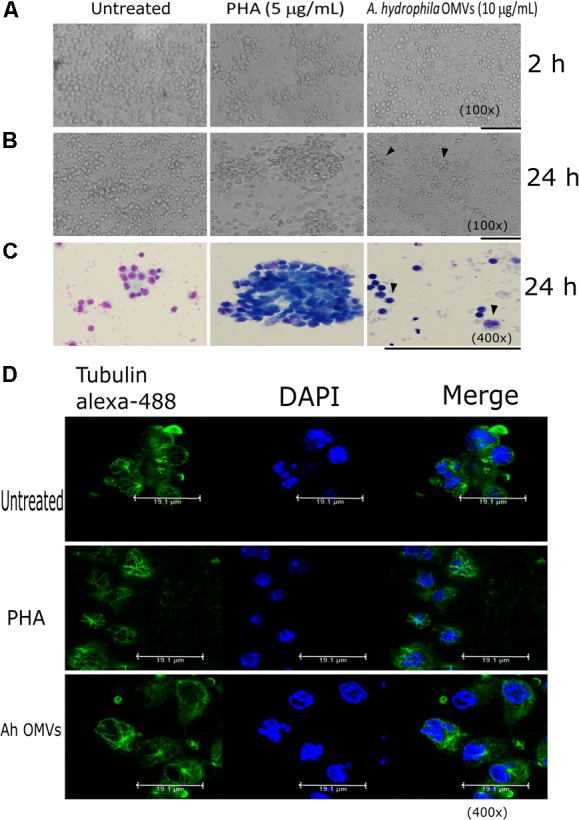
Morphological alterations induced by *A. hydrophila* ATCC^®^ 7966^TM^ OMVs in PBMCs. **(A)** After 2 h post-stimulation with *A. hydrophila* OMVs, no morphology changes of the PBMCs were observed compared with PBMCs after 24 h post-stimulation with vesicles, after 24 h post-stimulation conglomerated cells were observed (arrowheads) **(B)**. Giemsa staining revealed differences in the affinity of the dye to the nucleus and cytoplasm, bigger cells, and vacuolization of the stimulated PBMCs with *A. hydrophila* OMVs (arrowheads) **(C)**. Confocal microscopy, showed defective tubulin polymerization in PBMCs stimulated with OMVs **(D)**. bars = 50 μm.

*Aeromonas hydrophila* OMVs (10 μg/mL) induced activation of lymphocytes cells from PBMCs; cell activation induced changes in morphology and cytoskeleton organization. Therefore, we used 10 μg of OMVs to analyze morphology changes in PBMCs. PHA was used as positive control for cytoskeleton disruption, PHA is a lectin mitogen used to induce the immune response in leukocytes. PHA induces translocation of protein kinase C, Ca^2+^ movement, and IL-12 production which derivate in cytoskeleton rearrangement; cytoskeleton rearrangements are necessary for the signal transduction in the cells (Dupuis et al., 1993). After 2 h post-stimulation no changes in the morphology of the PBMCs were observed. However, after 24 h post-stimulation, the cells were enlarged and battered (Figure 7). Giemsa staining of the stimulated PBMCs with *A. hydrophila* OMVs showed differences in the affinity of the dye to the nucleus and cytoplasm, enlarged cells, and vacuolization inside some cells (Figure 7C). We analyzed by confocal microscopy the cytoskeleton structure of host cells with immuno-staining with mAb anti-tubulin observing microtubules organization in PBMCs stimulated with OMVs. This experiment revealed that *A. hydrophila* OMVs impairs organization and polymerization of microtubules compared with unstimulated cells (Figure 7). Alike with Giemsa staining, fluorescence of the nucleus with DAPI showed different staining patterns and morphological alterations.

## Discussion

In the last years OMVs have become an interesting bacterial model of study. Many functions have been awarded to these nanovesicles like to be a vehicle to transport virulence factors, in biofilm formation, in the stress response, as inducers of the immune response, among others (Pérez-Cruz et al., 2015). But also the OMVs have been used as platform for vaccine development (Roier et al., 2010; van de Waterbeemd et al., 2010; van der Pol et al., 2015).

Even though much information has been published about OMVs from several pathogens, due to the paucity of data on OMVs from *A. hydrophila*, in this work we aimed to analyze the protein composition of the OMVs purified from *A. hydrophila* ATCC^®^ 7966^TM^, as well as to evaluate the capacity to elicit immunological response and cytotoxic effect on host cell.

Some virulence factors have been associated to OMVs in pathogenic bacteria. Recently, it was reported that a Zn^++^ dependent hemagglutinin protease (HAP) was associated to OMVs of *V. cholerae* and induced dose dependent apoptosis in cells and enterotoxic response (Mondal et al., 2016). Also other enzymes have been associated to OMVs in *Vibrio*, and considered as important molecules in the interaction with the host cell (Rompikuntal et al., 2015). In the case of the OMVs from *Piscirickettsia salmonis*, a fish pathogen, induced a cytopathic effect on CHSE-214 cells, suggesting a role in pathogenesis (Oliver et al., 2016). Although *Aeromonas* spp. are closed related with genus *Vibrio* and some species could be involved in fish diseases, less is known about the presence of virulence factors in the OMVs and how they could influence the host-bacteria interaction.

It is well known that *A. hydrophila* secrete a wide array of extracellular enzymes, these include chitinases, lipases and amylases (Kidd and Pemberton, 2002). A putative pulallanase was found in purified vesicles from *A. hydrophila*, this is an amylase with activity on starch. Two α-type amylases were previously reported in *A. hydrophila* (Kidd and Pemberton, 2002). These enzymes cleave α-1,4- linkages of starches or polysaccharides releasing products for use by the cell. The OMVs are considered a new secretion system, that have the advantage to transport several proteins including virulence factors in the same vehicle. So, in the case of pulallanase, this enzyme could be secreted in absence of a secretory pathway but transported in the vesicles.

*Aeromonas* display a number of hemolysins, in the vesicles we identified the heat-labile hemolysin hh1, which has cytolytic activity to red cells (Hirono and Aoki, 1991). There is evidence which indicates that the proteins found in vesicles are selected, while others are excluded. However, the mechanism for the selection of the proteins is yet unknown.

A triacylglycerol lipase was found in the OMVs; lipases also are considered as virulence factor in *Aeromonas*. Two lipases have been previously described in *Aeromonas, apl*-1, and *lip*. The *A. hydrophila, lip* gene was cloned and expressed in *A. hydrophila* and *E. coli*, the recombinant protein showed lipolytic activity (Singh et al., 2013). Also, we identified the extracellular lipase encoded by the *alt* gene with a triglyceride lipase activity well known as a cytotonic heat-labile enterotoxin (Alt). Sha and coworkers demonstrated that this enterotoxin induced gastroenteritis in mouse model (Sha et al., 2002).

The peptidoglycan-associated lipoprotein also known as Pal its part of the Tol-Pal complex composed in most of the Gram-negatives by 5 main proteins: TolA, TolB, TolQ, TolR, and Pal. TolB, TolQ, and Pal were found in *Aeromonas* vesicles but not TolA, or TolR. Pal purified from *E. coli* induced immune response in macrophages and spleen lymphocytes with production of pro-inflammatory cytokines (Hellman et al., 2002). The presence of some parts of Tol-Pal complex in the *Aeromonas* OMVs is unknown.

The universal stress protein belonging to the A family was found in OMVs. This protein is predicted to interact with chaperone GroEL, hypothetical protein (246 aa) and Kef-type K+ transport system membrane protein (646 aa), however, these last 3 proteins were not found in OMVs.

Siderophores are Fe (III) specific ligands that scavenge environmental sources of iron and the iron-siderophore complex enters the host cell through an outer membrane receptor. The outer membrane receptor (AHA_3963), TonB-dependent siderophore receptor (AHA_1969), and enterobactin synthase subunit E (entE) are co-oexpressed and involved in siderophore function. These three proteins were found in vesicles of *A. hydrophila*. The Ton B-dependent receptor proteins were also found in vesicles from *Brucella*, an intracellular pathogen. Authors suggested that Ton B-dependent receptors could be involved in siderophore internalization and help in the survival in nutrient-limiting conditions such as found in macrophages (Avila-Calderón et al., 2012).

Through the study of the deletion mutant of *rtx*A1 gene in *V. vulnificus*, Kim and coworkers demonstrated that Rtxa1 toxin is a multifunctional cytotoxin. The Rtx toxin of *V. vulnificus* induced cytoskeletal rearrangements, caused hemolysis through pore formation, induced cytotoxicity and the toxin expression increased after host cell contact (Kim et al., 2008). In the case of *A. hydrophila*, RtxA was exclusively expressed and produced during co-infection with the host cells, as it was previously observed in *V. vulnificus* (Kim et al., 2008; Suarez et al., 2012). The RtxA toxin is transported by T1SS in *V. cholerae*, so the OMVs could be an alternative pathway to release this virulence factor.

OmpC is the third major porin in *E. coli*, showing a cation selective pore (Nikaido, 2003). Recently, a vaccine based on purified *A. hydrophila* OmpC was tested in *Cirrhinus mrigala* and then challenged with live *A. hydrophila.* The aim of such study was to evaluate if the pure OmpC could induce the expression of myxovirus protein genes in the carp. Authors found that OmpC of *A. hydrophila* could stimulate the antiviral Mx gene of *C. mringala* (Roy et al., 2016). In this sense, *Aeromonas* OMVs could be evaluated as platform to develop acellular vaccines.

Some authors have been described that two potent toxins like AexU and cytolytic toxin Act promote damage in host cells. AexU protein inhibits IL-6 and IL-8 secretion from HeLa cells (Sierra et al., 2010), while toxin Act induce cleavage of caspase-3, and mitogen-activated protein kinase (MAPK) pathways in human intestinal epithelial cells (Galindo et al., 2004).

Here, we demonstrated that *A. hydrophila* OMVs induce pro-inflammatory cytokine environment to stimulate human PBMCs with highest production of IL-8 and IL-1β.

Due to the nanostructure of the OMVs, these vesicles could reach tissue more deeply than whole cell. Also OMVs could spread far away from the site of infection, acting like a long-distance vehicle for delivering virulence factors. *Aeromonas* spp. infection may occur through the water and food leading to diarrheogenic symptoms and bacteremia in immunocompromised patients (Sebo et al., 2006). It is possible that during ingestion of contaminated food, some bacteria reach the regional lymph nodes, and encounter with lymphocytes and antigen presenting cells (APCs). Once established infection, *Aeromonas* spp. release OMVs which disseminate far from the local site, for instance, bloodstream and interact with PBMCs.

Direct stimulation of human PBMCs, could be used as *in vitro* model of intestinal inflammation caused by bacterial pathogens (Caballero and Pamer, 2015; Fábrega et al., 2016). The stimulation of PBMC with OMVs and formalin-killed *Aeromonas* (FKB) clearly induced different cytokine profiles such as the highest induction of IL-10, IL-6, and IL-8 by OMVs. This could be due to the presence of components such as FlgE and the glyceraldehyde-3-phosphate dehydrogenase in the OMVs. These secreted proteins help the pathogen establishing the infection and modulate the immune response (Aguilera et al., 2014; Fábrega et al., 2016). Clearly, the FKB and the OMVs signaling different pathways due to the different protein content and it is reflected in the cytokine production.

Stimulation of PBMCs with OMVs induced over-expression of early activation marker CD69 in LnT CD3+, and LnB CD19+, but not in monocyte population (CD91+). Under basal conditions, when peripheral blood lymphocytes are not stimulated, these cells do not express CD69 or express at low levels. Therefore, up-regulation of CD69 expression leads to induction of several cellular processes in lymphocytes like proliferation, calcium influx among others (Marzio et al., 1999). Despite that CD69 is not usually used as activation marker for peripheral monocytes, over-expression of this marker in monocytes is linked to 5-lipooxygenase and leukotrienes metabolism. Thus, CD69 over-expression in peripheral monocytes could indicate whether stimulation with *A. hydrophila* OMVs induce inflammatory process (Wöbke et al., 2013).

The costimulatory molecule CD86 (B7.2) is constitutive expressing in APCs such as dendritic cells, macrophages, monocytes, B cells, and up-regulating by activation in T cells (Wang and Chen, 2004). Raw OMVs induced over-expression of CD86 in CD19+ cells, but not in CD3+ cells. CD86 in B cells (CD19+) could be enhanced not only by T-B cell cooperation, but also by B cell receptor (BCR) simulation with OMVs antigens. It has been described a B cell population CD21^low^CD86^high^, which are potent APCs with great repertory of chemokine receptor molecules which allows this cells migrate to site of inflammation and leading immune response induction by Ag-presentation and cytokine production; these cells are called B_APC_. B_APC_ cells are originated from reacting B cells (B_react_ CD21+CD86+) after stimulation. After prolonged stimulation B_react_ decrease CD21 expression leading to B_APC_ phenotype (Shimabukuro-Vornhagen et al., 2017). We speculated that *A. hydrophila* OMVs induce a B_APC_-like phenotype after stimulation; our results show that B cells CD19+ express quite levels of CD86 expression previous OMVs stimulation corresponding to B_react_ phenotype. We not include CD21 in the characterization of B cells, however, Shimabukuro-Vornhagen et al. (2017) have been reported both populations of B cell in bloodstream also the authors report that B_react_ cells are recently activated B cells and B_APC_ phenotype is acquired after prolonged stimulation, and this change rely on the strength and duration of stimulation. We observed that over-expression of CD86 in B cells is significantly high at 24 and 48 h post-stimulation, we hypothesized that this corresponding an early stage of B_APC_ phenotype (Shimabukuro-Vornhagen et al., 2017).

We could not observe significant high levels of expression of CD86 and CD69 in monocytes, conversely expression of these surface molecules drop at 48 h post-stimulation. Although monocytes CD91+ acts as APCs up take and presenting Ags, and over-expressing co-stimulatory molecules such as CD86, lymphocytes are the major population in PBMCs, we speculate that OMVs interact mainly with T and B cells and induce over-expression of activation markers and co-stimulatory molecules in lymphocytes compared to a monocyte population.

PD-1 and their ligand PD-L1 are molecules members of the B7/CD28 superfamily, and are related with inhibitory signals in lymphoid and myeloid cells (Nurieva et al., 2009). Role of PD-L1 and PD-1 seems to be contradictory, some authors report that PD-1 and their ligand PD-L1 leads exhaustion of T cells, whereas others indicate that expression of these molecules not necessary leads to T cell dysfunction. For instance, infection with Friend virus (FV) in a mouse model, cells with high expression of PD-L1 avoid elimination from CD8+ T cells. Same authors reported similar effects in a CD4+ T cells infected with HIV; infected CD4+ T cells which express PD-L1 avoid cytotoxic responses (Akhmetzyanova et al., 2015). However, little is known about the participation of PD-1 and PD-L1 during the infection with bacterial pathogens, specifically extracellular bacteria like *Aeromonas*.

Stimulation of PBMCs with raw OMVs from *A. hydrophila* induced early expression of PD-1 mainly in LnT CD3+, whereas only the high doses of OMVs induce over-expression in B cells. It seems that PD-1 effect on T cells activation is not completely inhibitory, even during viral infection; i.e., Zelinskyy et al. (2011) found that PD-1 was up-regulated during FV acute infection and CD8+ T cells remain functional during the first two weeks. On the other hand, in mouse model it has been described that over-expression of PD-1 by conventional natural killer (NK) cells is required for protection against *Citrobacter rodentium* infection (Solaymani-Mohammadi et al., 2016). Here, PD-1 expression correlates with early activation marker CD69 in LnT CD3+ and inflammatory cytokine production like TNFα; PD-1 expression has significant effect on pro-inflammatory cytokines (Keir et al., 2008).

Moreover, over-expression of PD-L1 was observed in both population of lymphocytes. Although the expression of PD-L1 remained during all time points in T cells, in B cells dropped at late times. PD-L1 expression has been involved in bacterial clearance; i.e., Xu et al. (2013) reported that PD-L1 co-stimulate T cells CD8+ during *Listeria monocytogenes* infection and is required for bacterial clearance. In the same way, López-Medina et al. (2015a) observed that B cells co-expressed co-stimulatory molecules such as CD86 and PD-L1 at 3-day post-infection with *Salmonella typhimurium* as we shown here. Although Lopez-Medina et al., argued that PD-L1 expression in B cells and APC renders to long-lasting infection with *Salmonella* by CD8+ T cell dysfunction. The authors described a continuous activated state of the cells by the expression of co-stimulatory molecules, with a predominant PD-L1 inhibitory response which leads to T cells exhaustion (López-Medina et al., 2015a,b).

We suggest that PD-1 and PD-L1 over-expression induced by *A. hydrophila* OMVs correlate with higher levels of CD69 and CD86, and it is related to lymphocyte activation: (i) PD-1 expression is not sustained during all the time of experiment in T cells; (ii) T cells over-express early activation marker CD69; (iii) PD-L1 expression dropped in B cells at late time, whereas CD69 is sustained during all the time; (iv) while PD-L1 expression dropped at 48 h, CD86 is up-regulated at 48 h in B cells.

Therefore, the pro-inflammatory cytokine profile together with lymphocyte activation induced by OMVs could contribute with the pathogenesis of *Aeromonas* infection. For instance, immunocompromised patients could develop sepsis by bacterial translocation (*Aeromonas* septicemia) (Sebo et al., 2006). In the bloodstream, OMVs activate lymphocytes and induce cytokine production such as IL-6, IL-1β, and IL-8. Pro-inflammatory cytokines activate endothelium and are chemoattractant for polymorphonuclear cells which promote destructive inflammation in different tissues (Park et al., 2010).

We could not observe monocyte activation or significant expression of co-stimulatory molecules, in contrast we observed high expression of cleaved PARP in cells from the monocytic region. Based on these results we speculate that OMVs stimulation impairs monocyte function. Once monocytes phagocytized, OMVs allow the entry of virulence factors, then higher doses of virulence factors lead apoptosis and drive the expression of cleaved PARP.

As we aforementioned, *A. hydrophila* RtxA induced apoptosis and cell rounding in HeLa cells by rearrangement of actin cytoskeleton (Suarez et al., 2012). Therefore, we decided to explore the effect of the OMVs on the cytoskeleton rearrangement by phalloidin staining for actin-microfilaments and using monoclonal antibodies against α-tubulin. However, no significant rearrangement or depolimerization of actin was observed (Supplementary Figure S2) compared with the changes in the tubulin-microtubules. This result could be attributed to the content of the OMVs. In *A. hydrophila* OMVs were found some proteins of the machinery of the Type 6 secretion system (T6SS) such as Hcp and putative T6SS-proteins like AHA_1832 that could be involved in the tubulin rearrangement. Previously, the VgrG1 a protein effector of the type VI secretion system (T6SS) was identified in *A. hydrophila*. The expression of VgrG1 in HeLa Tet-Off cells disrupted the actin cytoskeleton, decreased the cell viability and increased the apoptosis (Suarez et al., 2010). Further investigation should be done to determine if the putative T6SS-proteins found in the OMVs are related somehow in the rearrangements of the cytoskeleton of host cell.

In this work we demonstrated that OMVs from *A. hydrophila* 7966 carry out a variety of proteins including virulence factors. Also that these vesicles were cytotoxic for PBMCs, induced activation of B cells and rearrangement of tubulin cytoskeleton.

## Author Contributions

EA-C, JO-O, AC-R, and LF-R conceived and designed the experiments. EA-C, JO-O, AC-R, and JC-A performed the experiments. EA-C, HP, OM-C, and LD-M analyzed the data. LF-R and MA-A gave practical suggestions to perform experiments. EA-C, MA-A, HP, MM-G, MR-P, and AC-R wrote the paper.

## Conflict of Interest Statement

The authors declare that the research was conducted in the absence of any commercial or financial relationships that could be construed as a potential conflict of interest.

## Footnotes

1 http://www.ncbi.nlm.nih.gov/nuccore/117617447

2 http://www.psort.org/psortb/index.html

3 http://linux1.softberry.com/berry.phtml?topic=pcompb&group=programs&subgroup=proloc

## Supplementary Material

FIGURE S1 Enrichment of function for proteins identified in *Aeromonas hydrophila* ATCC^®^ 7966^TM^ OMVs. According to the COG annotation, proteins identified in OMVs **(A)** were grouped into (i) Information, storage and processing proteins (translation, ribosomal structure and biogenesis, RNA processing and modification, transcription, replication, recombination and repair, chromatin structure and dynamics), cellular processing and signaling proteins (cell cycle, cell division, chromosome portioning, defense mechanisms, signal transduction mechanism, cell wall/membrane/envelope biogenesis, cell motility, intracellular trafficking, secretion, and vesicular transport, posttranslational modification, protein turnover, chaperones), (ii) metabolisms proteins (energy production and conversion, carbohydrate transport and metabolism, amino acid transport and metabolism, nucleotide transport and metabolism, coenzyme transport and metabolism, lipid transport and metabolism, inorganic ion transport and metabolism, secondary metabolites biosynthesis, transport and catabolism), (iii) and poorly characterized proteins (general function prediction only, function unknown, not COG annotation). Clearly, a great variety of protein related-functions were enriched in OMVs compared with *A. hydrophila* genome **(B)**.

FIGURE S2 Effect on the actin rearrangement induced by *A. hydrophila* OMVs in PBMCs. Compared with the PHA control, OMVs from the *A. hydrophila* ATCC^®^ 7966^TM^ does not induce deposition or depolimerization of actin-microfilaments.

TABLE S1 Proteins identified in OMVs from *A. hydrophila* 7966.
