# Supplementary figures and images for: The Outer Membrane Vesicles of Aeromonas hydrophila ATCC® 7966TM: A Proteomic Analysis and Effect on Host Cells
Source: Front Microbiol. 2018 Nov 16;9:2765. doi: 10.3389/fmicb.2018.02765 (PMC6250952; doi:10.3389/fmicb.2018.02765)

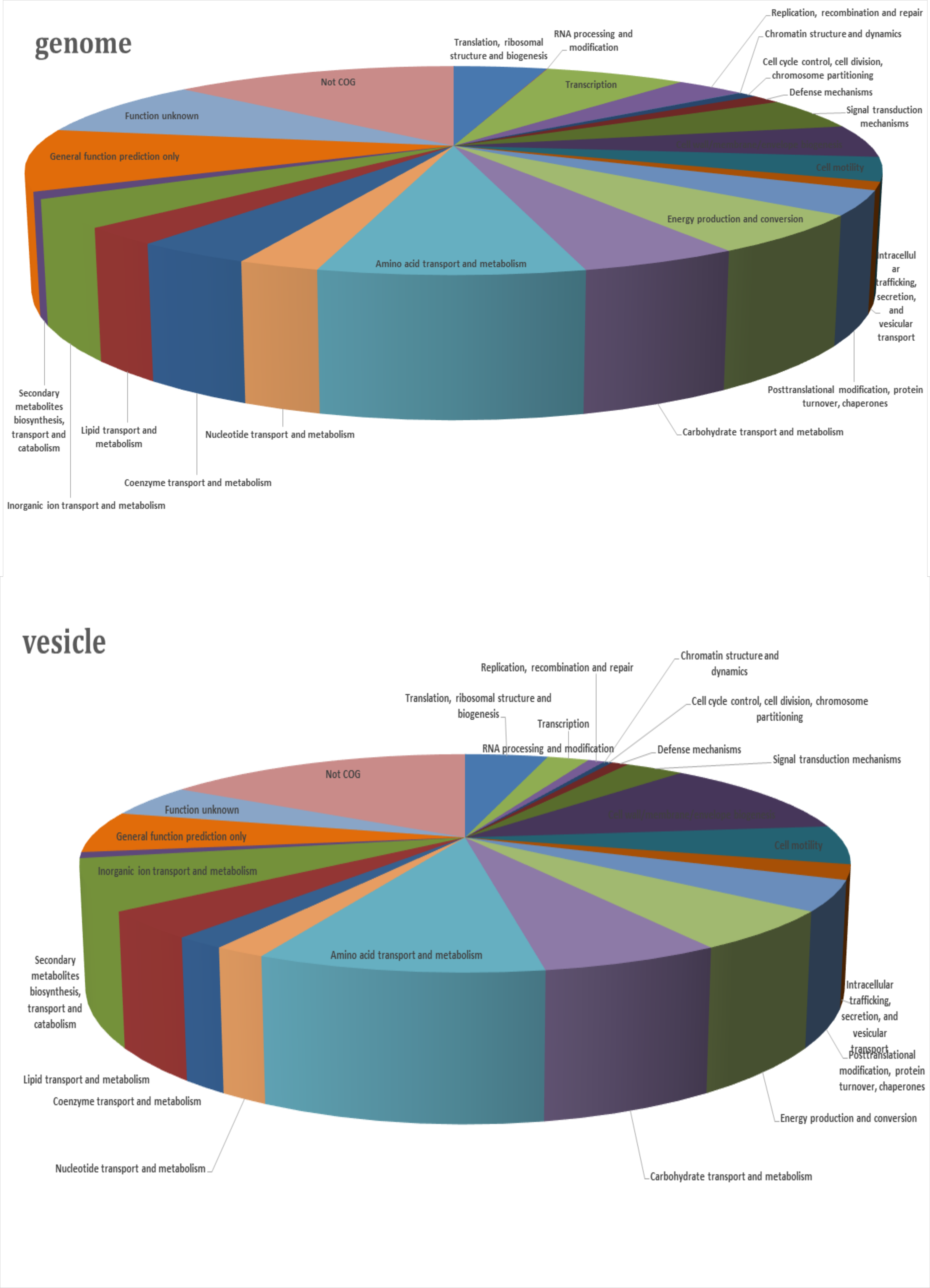

Supplement: FIGURE S1 — Enrichment of function for proteins identified in Aeromonas hydrophila ATCC® 7966TM OMVs. According to the COG annotation, proteins identified in OMVs (A) were grouped into (i) Information, storage and processing proteins (translation, ribosomal structure and biogenesis, RNA processing and modification, transcription, replication, recombination and repair, chromatin structure and dynamics), cellular processing and signaling proteins (cell cycle, cell division, chromosome portioning, defense mechanisms, signal transduction mechanism, cell wall/membrane/envelope biogenesis, cell motility, intracellular trafficking, secretion, and vesicular transport, posttranslational modification, protein turnover, chaperones), (ii) metabolisms proteins (energy production and conversion, carbohydrate transport and metabolism, amino acid transport and metabolism, nucleotide transport and metabolism, coenzyme transport and metabolism, lipid transport and metabolism, inorganic ion transport and metabolism, secondary metabolites biosynthesis, transport and catabolism), (iii) and poorly characterized proteins (general function prediction only, function unknown, not COG annotation). Clearly, a great variety of protein related-functions were enriched in OMVs compared with A. hydrophila genome (B). [file Image_1.tif]

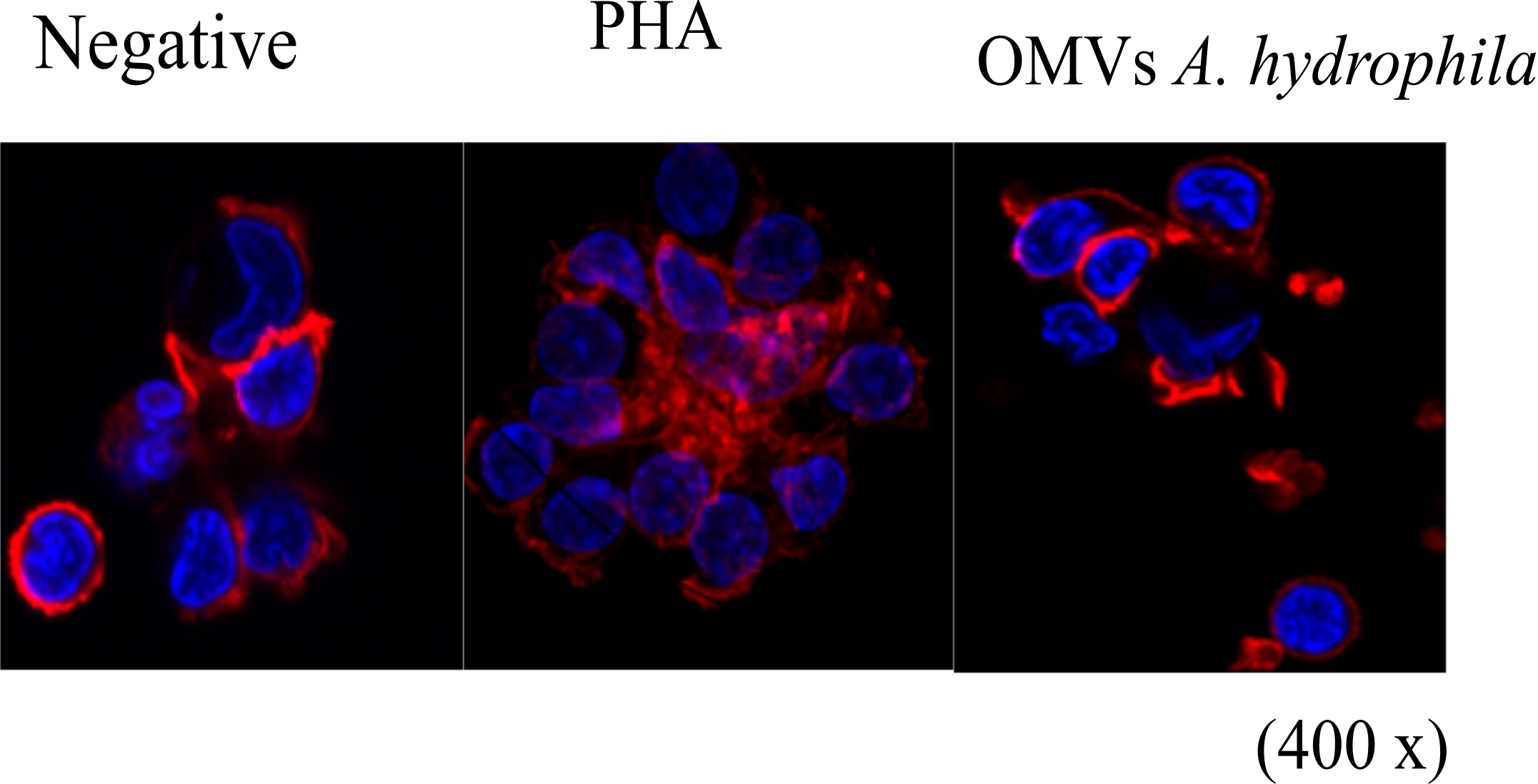

Supplement: FIGURE S2 — Effect on the actin rearrangement induced by A. hydrophila OMVs in PBMCs. Compared with the PHA control, OMVs from the A. hydrophila ATCC® 7966TM does not induce deposition or depolimerization of actin-microfilaments. [file Image_2.tif]
